# A Prospective Randomized Trial: Does Full Weight Bearing Improve Functional Outcomes in Elderly Patients With Proximal Intramedullary Nailing After an Intertrochanteric Femur Fracture?

**DOI:** 10.7759/cureus.48997

**Published:** 2023-11-18

**Authors:** Duran Topak, Mustafa Abdullah Özdemir, Mikail Telek, Sefa Kaya, İbrahim Halil Yönder, Bora Bilal, Fatih Doğar, Okkes Bilal

**Affiliations:** 1 Orthopedics and Traumatology, Kahramanmaraş Sütçü Imam University Medical Faculty, Kahramanmaraş, TUR, Kahramanmaraş, TUR; 2 Orthopedics and Trauma, Kahramanmaraş Sütçü Imam University Medical Faculty, Kahramanmaraş, TUR; 3 Orthopedics and Traumatology, Kahramanmaraş Sütçü Imam University Medical Faculty, Kahramanmaraş, TUR; 4 Anesthesia and Critical Care, Kahramanmaraş Sütçü Imam University Medical Faculty, Kahramanmaraş, TUR; 5 Orthopedics and Traumatology, Kahramanmaraş Sütçü Imam University, Kahramanmaraş, TUR

**Keywords:** barthel’s index of activities of daily living, weight bearing, harris hip score, femur intertrochanteric fracture, elderly hip fracture

## Abstract

Introduction

This study aimed to compare the functional outcomes and degree of independence in activities of daily living in patients aged >65 years who were treated with a proximal femoral nail (PFN) after an intertrochanteric femur fracture (ITFF) and underwent full and partial load-bearing in the early stage.

Methods

Overall, 133 patients who were hospitalized for ITFF and treated with PFN between August 2018 and March 2021 were randomly assigned to two groups. During the follow-up period, 45 patients who underwent partial load bearing (Group 1) and 40 patients who underwent full load bearing (Group 2) were prospectively evaluated. The Harris hip score was used for functional evaluation, and the Barthel index was used to evaluate the degree of independence in activities of daily living.

Results

The mean age of the patients included in the study was 76.67 ± 8.62 years. Regarding the comparison among groups in terms of age, sex, direction of fracture, reduction quality, fracture type, tip-apex distance, and surgical risk, there was no statistically significant difference between the two groups (p ≥ 0.05). Moreover, regarding the comparison in terms of calcium, phosphate, alkaline phosphatase, vitamin D, and keratin levels, which affect bone metabolism, no statistical difference was observed (p ≥ 0.05). We found that the mean Harris hip score was significantly higher in Group 1 than in Group 2 (Group 1: 76.82 ± 12.48; Group 2: 67.80 ± 15.34; p = 0.004). Moreover, 73.3% (n=33) and 42.5% (n=17) of patients in Groups 1 and 2 were fully independent or mildly dependent, respectively. We also found that the independence status was significantly better in Group 1 (p = 0.004).

Conclusion

Mobilization of older patients treated with PFN after ITFF using partial load-bearing protocols in the early postoperative period positively impacts hip function and the ability to perform activities of daily living independently.

## Introduction

Similar to all low-energy fractures, hip fractures are the most devastating osteoporotic fractures responsible for increased mortality in older men and women [1]. Notably, intertrochanteric femoral fractures (ITFF) account for approximately 50% of all hip fractures [2]. These fractures can be associated with complications such as disability, reduced quality of life, chronic pain, and death. With an aging population and constantly increasing life expectancy, the number of frail older adults at risk of falls and fractures is expected to increase. In addition, the burden of hip fractures - possibly associated with morbidity and dependency - is predicted to increase.

ITFF has been traditionally treated with extramedullary implants such as dynamic hip screws. Furthermore, cephalomedullary nails have become popular in the treatment of ITFF over the past two decades owing to significant advantages such as better biomechanical stability, minimally invasive surgery, short operating time, reduced blood loss, short hospitalization time, and early mobilization [3-5]. Notably, infection and nonunion rates are lower in patients treated with cephalomedullary nails [5].

To date, there has been no consensus among orthopedic surgeons about how much loading should be allowed in patients after surgical treatment of ITFFs. Although some surgeons report that partial load bearing delays functional recovery and the return to independent living in older patients, some surgeons avoid full load bearing because of complications that can arise from indefinite loading in the early stages.

This study aimed to compare the clinical and functional outcomes of patients aged >65 years treated with a cephalomedullary nail after ITFF and subjected to full and partial load bearing in the early phase; we also evaluated the effects of full load bearing on hip function.

## Materials and methods

Ethical approval for this randomized prospective study was obtained from the local ethics committee (date: 07.13.2018, session number: 12, decision number: 16). The aim of the study was explained to all patients, and informed consent was obtained from all participants. This study was registered in the Clinical Trials.gov PRS database (Clinical Trials.gov ID: NCT05602155).

Patients admitted to our hospital’s emergency department for ITFF and scheduled for treatment with a cephalomedullary nail were randomized into two groups by the hospital’s treating physician. In particular, surgeries were performed on all patients using the same technique and implant and by the same surgical team. Patients in Group 1 were postoperatively requested to walk with a load restriction of 10-20% of their body weight, and patients in Group 2 were requested to walk without any load restriction. In addition, patients were postoperatively taught the appropriate walking protocol with the aid of a walker by the ward physician.

Starting from the first postoperative day, the doctor in charge of the service taught him to walk with the help of a walker at a rate of approximately 10-20% of his body weight. The compliance of the patients was checked by the relevant doctor by gait evaluation during outpatient clinic visits. Partial load was started on the first day after surgery, and full load was started at the end of 6 weeks. All patients included in the study performed their daily living activities independently and did not need a walker or cane for mobilization.

 Overall, 133 patients who were hospitalized for ITFF between August 2018 and March 2021 were included in the present study. In contrast, patients with communication problems after surgery (n = 13), those who did not attend regular follow-up for 1 year (n = 12), those who died within the first year (n = 9), those who could not walk before surgery (n = 6), those who suffered from conditions affecting walking (cerebrovascular disease and fracture formation) within the first year (n = 5), and those who underwent hemiarthroplasty during surgery (n = 3) were excluded from the study. Demographic data and X-ray images of 45 patients who underwent partial load bearing (Group 1) and 40 patients who underwent full load bearing (Group 2) were obtained from electronic patient files.

The demographic data of the patients included in the study were obtained from medical records. The fracture classification was made based on the radiographic images of the patients according to the Evans-Jensen and OTA/AO classifications [6,7]. According to the AO classification revised in 2018, 31A1.1, 31A1.2, and 31A1.3 fractures are defined as stable fractures [7]. The surgical risks of the patients were evaluated according to the criteria of the American Society of Anesthesiology (ASA) [8]. Notably, fracture reduction was defined as good, acceptable, and poor according to the modified Baumgaertner reduction criteria, which was defined by Baumgaertner and modified by Fogagnolo [9]. Furthermore, fracture union was radiologically defined as callus formation and reduction of pain in the groin.

Post-treatment clinical evaluations were performed at the end of the six-month follow-up via face-to-face questionnaires and in the presence of relatives. The Turkish version of the Harris hip score, which has shown adequate internal consistency (Cronbach’s alpha, 0.70) and test-retest reliability (intraclass correlation coefficient (ICC) = 0.91) for functional outcomes [10], was used in the study. The Turkish version of the Barthel index developed by Mahoney and Barthel in 1965 and adapted by Küçükdeveci et al. was used to assess the degree of independence in activities of daily living. The score that can be obtained from the Barthel index ranges between 0 and 100, and higher scores indicate more independence in activities of daily living. The scores of 0-20, 21-61, 62-90, 91-99, and 100 indicated that the patient was fully dependent, highly dependent, moderately dependent, mildly dependent, and fully independent, respectively [11].

Statistical analysis

Statistical Package for Social Sciences Version 25.0 (IBM Corp., Armonk, NY) was used for data analysis. Number (n), percentage (%), and mean ± SD (Min-Max) were used for the evaluation of descriptive statistics. Kolmogorov-Smirnov test was used to determine whether the data conformed to the normal distribution. Homogeneity was evaluated using Levene’s test. Parametric tests were used for normally distributed measurements, and nonparametric tests were used for non-normally distributed measurements. Regarding the evaluation of differences between the groups, the independent samples t-test was used for normally distributed variables, whereas the Mann-Whitney U test was used for non-normally distributed variables. The relationship between two independent categorical variables was analyzed using the chi-square test. A p-value of <0.005 was considered statistically significant in all analyses.

## Results

The mean age of the participants was 76.67 ± 8.62 years. The patients were preoperatively hospitalized for an average of 1.74 days and a total of 4.49 days. Notably, the average hospitalization in the postoperative intensive care unit was 0.71 days. The mean Harris hip score of the patients was 72.57 ± 14.53 (32-91). The most common complication was dislocation of the screw from the femoral head; it was observed in four (4.7%) cases. Demographic characteristics of the patients are presented in Table 1.

**Table 1:** Distribution of the variables used in the research

Variables			Mean±SD (Min-Max), n (%)
Age (Year)			76.67±8.62 (65-93)
Gender		Female	42 (49.4%)
		Male	43 (50.6%)
Side		Left	41 (48.2%)
		Right	44 (51.8%)
Reduction quality		Good	72 (84.7%)
		Acceptable	10 (11.8%)
		Poor	3 (3.5%)
Evans classification (Grade I/II/III/IV/V)			17/9/8/26/25
AO Classification	A11		12 (14.1%)
	A12		17 (20.0%)
	A13		6 (7.1%)
	A21		9 (10.6%)
	A22		17 (20.0%)
	A23		3 (3.5%)
	A31		6 (7.1%)
	A32		4 (4.7%)
	A33		11 (12.9%)
Hospitalization (Day)			4.49±1.92 (2-10)
Harris hip score			72.58±14.54 (32-91)
Union time (Week)			8.84±2.21 (6-16)
Follow-up time (Month)			20.27±6.35 (12-34)
Barthel Index	Fully dependent		14 (16.5%)
	Highly dependent		21 (24.7%)
	Mildly dependent		10 (11.8%)
	Fully independent		40 (47.1%)
Complications	Cut out		4 (4.7%)
	Z effect		2 (2.4%)
	Implant failure		1 (1.2%)
	Infections		1 (1.2%)

Regarding the comparison between Groups 1 (partial load, n = 45) and 2 (full load, n = 40) in terms of age, sex, direction of the fracture, reduction quality, fracture type, tip-apex distance, and surgical risk, there was no statistically significant difference between the two groups (p > 0.05). When the two groups were compared in terms of calcium, phosphate, alkaline phosphatase, vitamin D, and keratin levels, which affect bone metabolism, no statistical difference was observed (p > 0.05). A comparison of the data between the two groups is presented in Table 2.

**Table 2:** Comparison of groups

Variables		Group 1 (n=45)	Group 2 (n=40)	p Value
Age (month)		76,58±9,38	76,77±7,79	0,917^*^
Gender	Female	22	20	0,919^**^
	Male	23	20	
Side	Left	23	18	0,574^**^
	Right	22	22	
Reduction quality	Good	38	34	0,718^**^
	Acceptable	6	4	
	Poor	1	2	
Fracture type	Stable	14	12	0,912^**^
	Unstable	31	28	
Surgical risk analysis	ASA-2	11	9	0,850^**^
	ASA-3	31	27	
	ASA-4	3	4	
Calcium (mg\dL)		7,78±0,76	8,84±0,66	0,709^*^
Phosphate (mg\dL)		3,01±0,81	3,09±0,79	0,653^*^
Alkaline phosphatase (U\L)		83,17±39,13	81,92±27,86	0,867^*^
Creatine (mg\dL)		0,92±0,43	0,82±0,27	0,200^*^
Vitamin D (µg\L)		8,11±6,95	10,77±8,15	0,108^*^
Complications	-	43	34	0,096^**^
	+	2	6	
Union time (week)		9,02±2,13	8,65±2,30	0,441^*^
Tip-apex distance (cm)		2,11±0,43	2,06±0,29	0,546^*^
Harris hip score		76,82±12,48	67,80±15,34	0,004^*^
Follow-up (month)		20,78±6,40	19,70±6,33	0,438^*^
Barthel Index	Fully dependent	7	7	0,004^**^
	Highly dependent	5	16	
	Mildly dependent	9	1	
	Fully Independent	24	16	
ASA; American Society of Anesthesiology ^*^Independent t test, ^**^chi-square test, p<0.05 statistically significant				

Although there were two (4.4%) and six (15%) cases of complications in Groups 1 and 2, respectively, the difference was not statistically significant (p = 0.096). Moreover, there was no significant difference between the union times of the two groups (Group 1 = 9.02, Group 2 = 8.65; p = 0.441).

Regarding the comparison of functional outcomes of the groups, the mean Harris hip scores were 76.82 ± 12.48 and 67.80 ± 15.34 in Groups 1 and 2, respectively. Moreover, the mean Harris hip score was significantly higher in Group 1 than in Group 2 (p = 0.004). Regarding the evaluation of independence status in activities of daily living, 73.3% (n = 33) of patients in Group 1 were fully independent or mildly dependent, whereas only 42.5% (n=17) of patients in Group 2 were fully independent or mildly dependent. Furthermore, we found that the independence status was significantly better in Group 1 (p = 0.004). A comparison of the data between the two groups is presented in Table 2.

## Discussion

The primary aims of treatment in older patients with a proximal femoral fracture are pain relief, early mobilization, and maintenance of an independent lifestyle. In patients with a hip fracture, delay in getting out of bed has been shown to worsen early hip function and six-month survival [12]. Another study reported that the most important predictor of mortality in patients undergoing hip fracture surgery was the inability to stand, sit, or walk within two weeks of surgery [13]. Early mobilization and regaining the pre-fracture level of function as soon as possible after ITFF treatment is highly important. Early mobilization in such cases has been shown to be one of the most effective ways to prevent complications and reduce mortality [14,15]. However, if patients are exposed to the load too early, good outcomes can be compromised by the loss of reduction or failure of fixation, and reoperation may be required [16].

Although there is consensus among orthopedic surgeons about early mobilization in older cases of ITFF fixed with a proximal femoral nail (PFN), there is no consensus about the amount of load that must be borne by the involved extremity. In particular, loading protocols should maintain or accelerate fracture healing within physiological limits while preventing the loss of reduction or implant failure. Biomechanical and animal studies suggest that early loading is beneficial; however, clinical studies with a high level of evidence comparing loading protocols after ITFF are limited [17,18]. The present randomized prospective study was conducted to fill this gap in the literature.

The most important finding of our study is that in older patients treated with PFN after ITFF, partial loading protocols resulted in better hip function in the early post-treatment phase compared with full loading protocols. Another important finding is that the patients who underwent partial load bearing in the early post-treatment period were more independent in performing activities of daily living and were less dependent on the environment than patients who underwent full load bearing.

Furthermore, complications were less common in patients who were given a partial load in the early postoperative period compared with patients who were given a full load; however, the difference was not statistically significant. This may be attributed to the small number of participants in the study group.

One study reported that two of three patients with advanced-age ITFF present with pain and/or functional limitations lasting ≥6 months [19,20]. Given the negative impact of pain on function, improvements in pain management will certainly have a positive impact on orthogeriatric care in the future. Moreover, considering that each person has a different pain threshold and response, we believe that partial loading in osteoporotic bones fixed with PFN has a positive effect on hip function and independence in activities of daily living because of micromovement-induced pain in the fracture line as a result of full loading.

Animal osteotomy models have reported that controlled or moderate axial loading of the osteotomy site typically results in greater callus volume and faster union time compared with no loading or excessive early loading [21]. However, the results of the present study indicate that there is no significant difference in the union times between partial and full loading in the early phase in older ITFF patients treated with PFN.

The strength of the study is its randomized and prospective design. However, the limitations include the small number of cases, the lack of body weight and body mass index measurements, and the lack of an objective assessment of the load level of the involved extremity. Another important limitation is that bone mineral density was not assessed.

## Conclusions

In conclusion, the results of the present study eliminate uncertainty about the loading of the involved extremity in the early postoperative period in older patients undergoing fracture fixation with PFN after ITFF. We found that mobilization of older patients treated with PFN after ITFF using partial load-bearing protocols in the early postoperative period positively impacts hip function and the ability to independently perform activities of daily living. In order to objectively assess the load transferred by the patient to the involved extremity, further studies should be performed in which the total load transferred is measured using a continuous sole force sensor.
